# Big data analysis of treatment patterns and outcomes among elderly acute myeloid leukemia patients in the United States

**DOI:** 10.1007/s00277-015-2351-x

**Published:** 2015-03-20

**Authors:** Bruno C. Medeiros, Sacha Satram-Hoang, Deborah Hurst, Khang Q. Hoang, Faiyaz Momin, Carolina Reyes

**Affiliations:** 1 Stanford University, 875 Blake Wilbur Dr, Stanford, CA USA; 2 Q.D. Research, Inc., 8777 Auburn Folsom Road C501, Granite Bay, CA 95746 USA; 3 Genentech, Inc., 1 DNA Way, South San Francisco, CA USA; 4 University of California, San Francisco, San Francisco, CA USA

**Keywords:** Acute myeloid leukemia, Elderly, Treatment, Chemotherapy, Stem cell transplantation, Survival

## Abstract

Over half of patients diagnosed with acute myeloid leukemia (AML) are 65 years or older. We examined patient characteristics, treatment patterns, and survival among elderly patients in routine clinical practice. We utilized a retrospective cohort analysis of first primary AML patients in the linked Surveillance, Epidemiology, and End Results (SEER)-Medicare database. Patients were diagnosed between January 1, 2000 and December 31, 2009, >66 years, and continuously enrolled in Medicare Part A and B in the year prior to diagnosis. Kaplan-Meier curves and Cox proportional hazards regression assessed overall survival by treatment. There were 3327 (40 %) patients who received chemotherapy within 3 months of diagnosis. Treated patients were more likely younger, male, and married, and less likely to have secondary AML and poor performance indicators and comorbidity score compared to untreated patients. In multivariate survival analysis, treated patients exhibited a significant 33 % lower risk of death compared to untreated patients. Significant survival benefits were noted with receipt of intensive and hypomethylating agent (HMA) therapies compared to no therapy. A survival benefit with allogeneic hematopoietic stem cell transplantation was seen in younger Medicare patients. This real-world study showed that about 60 % of elderly AML patients remain untreated following diagnosis. Use of anti-leukemic therapy was associated with a significant survival benefit in this elderly cohort.

**Electronic supplementary material:**

The online version of this article (doi:10.1007/s00277-015-2351-x) contains supplementary material, which is available to authorized users.

## Introduction

A disproportionate number of newly diagnosed acute myeloid leukemia (AML) occurs in elderly patients, and it is also the leading cause of mortality from leukemia in the USA [1, 2]. The median age at diagnosis is 66 years, and incidence increases with age with over half of the patients diagnosed at age 65 or older[3]. The use of chemotherapy has increased over time but fewer than half of elderly patients receive anti-leukemic therapy and their outcomes remain dismal [4–6]. After successful induction of remission, disease relapse is inevitable in the majority of cases without and despite additional post-remission therapy [7]. Without treatment, patients succumb to their illness within weeks to months of diagnosis [8].

Treatment efficacy and tolerability have been shown to deteriorate markedly with age [4]. Conventional chemotherapy treatments for AML may be highly toxic, usually requiring prolonged inpatient treatment [6]. For this reason, the NCCN guidelines describe separate treatment recommendations for patients older than 60 years based on performance status, cytogenetic or molecular mutation, and comorbid conditions rather than relying on chronologic age alone [7]. For medically fit older adults, treatment with a combination of an anthracycline and standard dose cytarabine is recommended rather than other chemotherapy regimens or supportive care alone. For older adults with poor physical function and/or unfavorable risk disease, supportive care alone or less intensive chemotherapy with DNA hypomethylating agents or low-dose cytarabine is recommended. The use of allogeneic hematopoietic stem cell transplantation (HSCT) is considered a potential cure for AML, but its use is limited in older patients because of significant baseline comorbidities and increased transplant-related morbidity and mortality [9, 10]. Nonetheless, data from the Swedish Acute Leukemia Registry show that most patients up to 80 years actually tolerate and benefit from intensive treatment, despite deteriorating organ function [11, 12].

Although patients 65 years or older represent the majority of patients with cancer in the USA, a minority of them are enrolled in randomized clinical trials (RCTs). In fact, several studies have shown that only 1– % of elderly cancer patients are participating in clinical trials, thus providing a limited evidence base in which to evaluate treatment efficacy and safety in this population [13–15]. The most frequently cited factors for clinical trial ineligibility were advanced age or the presence of significant comorbidity [16]. In order to address the need for additional data in this population, given the limited clinical trial participation, the increased incidence of AML due to the aging population, and the limited treatment options, we used population-based data to examine Medicare beneficiaries following their diagnosis of AML. The information obtained provides an important context for identifying opportunities to improve the quality of treatment strategies and evaluate the benefits of new treatments under investigation.

## Methods

### Data sources

Patients were identified from the linkage of two data sources, the Surveillance, Epidemiology, and End Results (SEER) program database from the National Cancer Institute and the Medicare enrollment and claims files from the Centers for Medicare and Medicaid Services. Details of the linked SEER-Medicare database have been published elsewhere [17]. Briefly, the database combines clinical, demographic, cancer diagnosis, and cause of death information with claims data for adults age 65 and older enrolled in Medicare Parts A and B. SEER is a nationally representative collection of 18 population-based registries of all incident cancers from diverse geographic areas covering approximately 26 % of the US population. All incident cancer patients reported to the SEER registries are cross-matched with a master file of Medicare enrollment [18]. All Medicare beneficiaries receive Part A coverage (inpatient care, skilled nursing, home health care, and hospice care). Approximately 95 % of beneficiaries subscribe to Part B, which covers physician services and outpatient care. The SEER-Medicare linkage included all Medicare eligible persons appearing in the SEER data through 2009 and their Medicare claims for Part A (inpatient) and Part B (outpatient and physician services) through 2010. Institutional review board approval was waived because the SEER-Medicare data lack personal identifiers.

### Study population

Patients were eligible for inclusion in the study if diagnosed with first primary AML between January 1, 2000 and December 31, 2009, at least 66 years of age, and continuously enrolled in Medicare Parts A and B in the 12 months prior to diagnosis. Patients were excluded if their date of death was recorded prior to or the same month as diagnosis, if they were enrolled in a health maintenance organization (HMO) at any time during the 12 months prior to diagnosis (because complete claims data were unavailable for these patients), and if they had two or more claims for chemotherapy prior to diagnosis (to ensure that the cases were previously untreated).

### Study variables

SEER program registries routinely collect data on patient demographics (age, race/ethnicity, residence, and socioeconomic status [income and education per census tract]); primary tumor site, tumor morphology, and stage at diagnosis; first course of treatment; and follow-up for vital status. AML diagnosis was based on the International Classification of Disease for Oncology (3rd edition, ICD-O-3) histology codes in the SEER data. Median annual household income at the census tract level, and percentage of adults aged 25 or older with at least some college education at the ZIP code level in the SEER data were used as a proxy for socioeconomic status.

Risk status in AML is based on cytogenetics and molecular abnormalities, which were not available in the SEER data. Prior myelodysplastic syndrome (MDS) or myeloproliferative neoplasm (MPN) that transforms into AML has also poor prognostic features and occurs more commonly among elderly patients [19]. In the absence of cytogenetic data, prior MDS or MPN was used as a proxy for high-risk patients and was identified using diagnosis codes in Medicare Parts A and B claims files prior to AML diagnosis. SEER also does not include measures of performance status, such as Eastern Cooperative Oncology Group. Instead, we used Medicare claims to identify several indictors of poor performance status (PPI) [20], including the use of oxygen and related respiratory therapy supplies, wheelchair and supplies, home health agency services, and skilled nursing facility services that occurred 12 months prior to AML cancer diagnosis.

To assess baseline comorbidity burden, we utilized the National Cancer Institute (NCI) comorbidity index [21] to identify the 15 non-cancer comorbidities from the Charlson Comorbidity Index [22]. The index accounts for the number and seriousness of the conditions and a higher score indicates a greater burden of comorbid disease. Diagnosis and procedure codes were identified from Medicare claims 1 year prior to diagnosis and must appear on at least two different claims that are more than 30 days apart to ensure that “rule out” diagnoses are not counted as comorbid conditions.

Chemotherapy administration was identified using International Classification of Disease (9th revision), Clinical Modification (ICD-9-CM) diagnosis codes and procedural codes, and Healthcare Common Procedural Coding System (HCPCS) “J” codes were used to identify the specific drug administered [23]. The absence of these claims indicated lack of treatment. The first chemotherapy claim within 3 months from diagnosis indicated the start of therapy. Patients were classified into treatment groups based on all chemotherapy administered during the first 60 days after treatment initiation. Chemotherapy agent definition was not possible in approximately 70 % of patients who received therapy because chemotherapy was administered during inpatient stays which are paid based on ICD-9 diagnosis or procedure codes only and not chemotherapy codes. Medicare claims files were also searched for ICD-9-CM and HCPCS codes to identify patients undergoing allogeneic HSCT anytime during follow-up.

Overall survival was measured from date of diagnosis to date of death. The date of death was assigned by using the Medicare date or SEER date of death if Medicare date was missing. All other patients were assumed to be alive at the end of the follow-up period (December 31, 2010), although they may have been censored earlier for other reasons such as the development of a second primary cancer or Medicare claims no longer available.

### Statistical analysis

All statistical analyses were performed using SAS software, version 9.1.3 (SAS Institute Inc., Cary, NC). Demographic and clinical characteristics were summarized descriptively by treatment status (treated vs. not treated) and treatment type. Chi-square test for categorical variables and ANOVA or *t* test for continuous variables determined differences between groups. We considered a *p* value < .05 to be statistically significant.

In the overall survival analyses, we made comparisons between the treated and not treated patients, between treated patients receiving HSCT and those who did not, and between those receiving low-dose therapy with a DNA methyltransferase (DNMT) inhibitor (azacitidine or decitabine (HMA therapy)), those receiving aggressive induction therapy (cytarabine + anthracycline (intensive therapy)), and those not receiving treatment. Kaplan-Meier survival curves and corresponding log rank tests examined unadjusted overall survival by treatment group. Since timing of treatment initiation differed between patients, the relationship between treatment and survival was evaluated using a Cox regression model with treatment as a time-dependent factor. In the time-varying Cox model, all patients belong to the “not treated” group and only switched to the “treated” group at the time of treatment receipt. Other confounders included in the Cox model were selected a priori from baseline demographic and clinical characteristics..

To assess the risk of early death (30-day mortality and 60-day mortality) after diagnosis, we also used a Cox regression model with treatment as a time-dependent factor. The treated group was limited to patients who received treatment within 30 days after diagnosis to minimize the introduction of immortal time bias in the analysis (period of follow-up time during which death cannot occur) [24].

As a sensitivity exercise for the comparison between HMA therapy, intensive therapy, and no treatment, we also conducted a propensity score-matched survival analysis. Multinomial logistic regression was used to calculate a propensity score—the conditional probability that each patient would be assigned to a specific treatment group given the patient’s pretreatment variables [25, 26]. Pairwise matching was conducted where each patient receiving HMA therapy was matched to one untreated patient and each patient receiving intensive therapy was matched to one untreated patient. Matching variables were age, sex, race, marital status, education, geographic region, year diagnosed, prior MDS, poor performance indicators, and comorbidity score. Matched survival analysis was completed using the Cox proportional hazards regression model, stratifying on the matched pair. Factors that were still found to be significantly different after matching (age, geographic region, and year diagnosed) were included as covariates in the Cox proportional hazards models.

In the survival models, follow-up was calculated beginning on the date of diagnosis up until the first occurrence of a censoring event: date of death, development of a second primary tumor, the last date for which Medicare claims are available, or the end of the follow-up period (December 31, 2010).

## Results

### Treatment trends over time

**Fig. 1:**
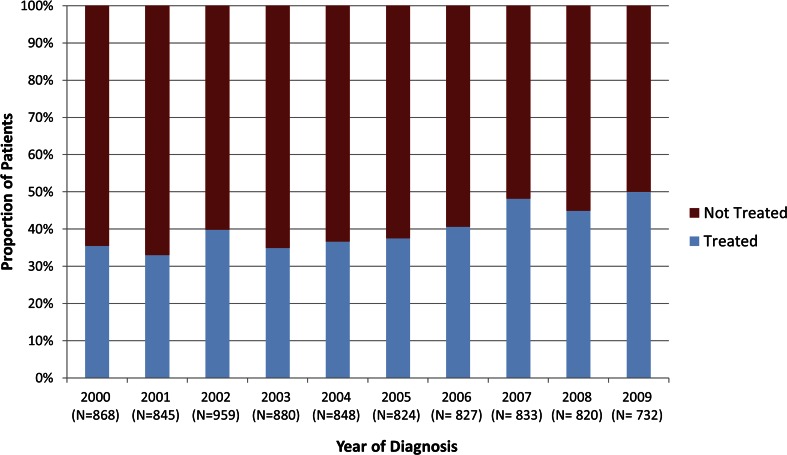
Treatment status by year of diagnosis

Of the 8336 patients who met all study criteria, 3327 (40 %) received treatment with chemotherapy within 3 months of diagnosis and 5009 (60 %) did not (Table 1). Treatment rates increased over the study time period from 35 % in 2000 to 50 % in 2009 (Fig. 1). Treated patients were younger at diagnosis with mean age of 75 compared to those not receiving treatment (81 years; *p* < .0001). Fifty-two percent of untreated patients were over the age of 80 compared to 20 % in the treated group (*p* < .0001). Treated patients were also more likely to be male (55 vs. 50 %), married (61 vs. 47 %), and have lower incidence of secondary AML (15 vs. 19 % prior MDS or MPN), were less likely to have PPI (7 vs. 17 %), and had lower comorbidity burden (*p* < .0001) than untreated patients.

**Table 1:** Baseline patient characteristics by treatment status

Characteristic	Total (*N* = 8336)		Treated (*N* = 3327)		Not treated (*N* = 5009)		*p* value
	*n*	%	*n*	%	*n*	%	
Age at diagnosis							
66–70	1322	15.9	881	26.5	441	8.8	<0.0001
71–75	1774	21.3	976	29.3	798	15.9	
76–80	1971	23.6	803	24.1	1168	23.3	
> 80	3269	39.2	667	20.0	2602	51.9	
Sex							
Male	4331	52.0	1832	55.1	2499	49.9	<0.0001
Female	4005	48.0	1495	44.9	2510	50.1	
Race/ethnicity							
White	7285	87.4	2918	87.7	4367	87.2	0.4807
Non-white	1051	12.6	409	12.3	642	12.8	
Prior MDS^a^							
No	6896	82.7	2839	85.3	4057	81.0	<0.0001
Yes	1440	17.3	488	14.7	952	19.0	
PPI^b^							
No	7280	87.3	3111	93.5	4169	83.2	<0.0001
Yes	1056	12.7	216	6.5	840	16.8	
NCI co-morbidity score							
0	4266	51.2	1899	57.1	2367	47.3	<0.0001
1	2104	25.2	842	25.3	1262	25.2	
2	1018	12.2	325	9.8	693	13.8	
≥ 3	948	11.4	261	7.8	687	13.7	
Marital status							
Married	4373	52.5	2028	61.0	2345	46.8	<0.0001
Widowed	2492	29.9	726	21.8	1766	35.3	
Separated/divorced	543	6.5	218	6.6	325	6.5	
Single	535	6.4	216	6.5	319	6.4	
Unknown	393	4.7	139	4.2	254	5.1	
% of adults with some college education							
0–50	3514	42.2	1370	41.2	2144	42.8	0.3260
51–100	4439	53.3	1799	54.1	2640	52.7	
Unknown	383	4.6	158	4.7	225	4.5	
Median income quartiles							
1–Low	2080	25.0	766	23.0	1314	26.2	0.0003
2	2080	25.0	819	24.6	1261	25.2	
3	2081	25.0	834	25.1	1247	24.9	
4–High	2079	24.9	902	27.1	1177	23.5	
Geographic region							
Midwest	856	10.3	377	11.3	479	9.6	0.0268
Northeast	517	6.2	216	6.5	301	6.0	
South	3136	37.6	1253	37.7	1883	37.6	
West	3827	45.9	1481	44.5	2346	46.8	

*NCI* National Cancer Institute, *MDS* prior myelodysplastic syndrome, *PPI* poor performance indicators

^a^Patients with a prior myelodysplastic syndrome (MDS) or myeloproliferative disease was identified from Medicare claims and was used as a proxy for high-risk patients in the absence of disease stage

^b^Poor performance indicators (PPI) were identified from Medicare claims and include the use of oxygen and related respiratory therapy supplies, wheelchair and supplies, home health agency services, and skilled nursing facility services that occurred 12 months prior to AML diagnosis

**Fig. 2:**
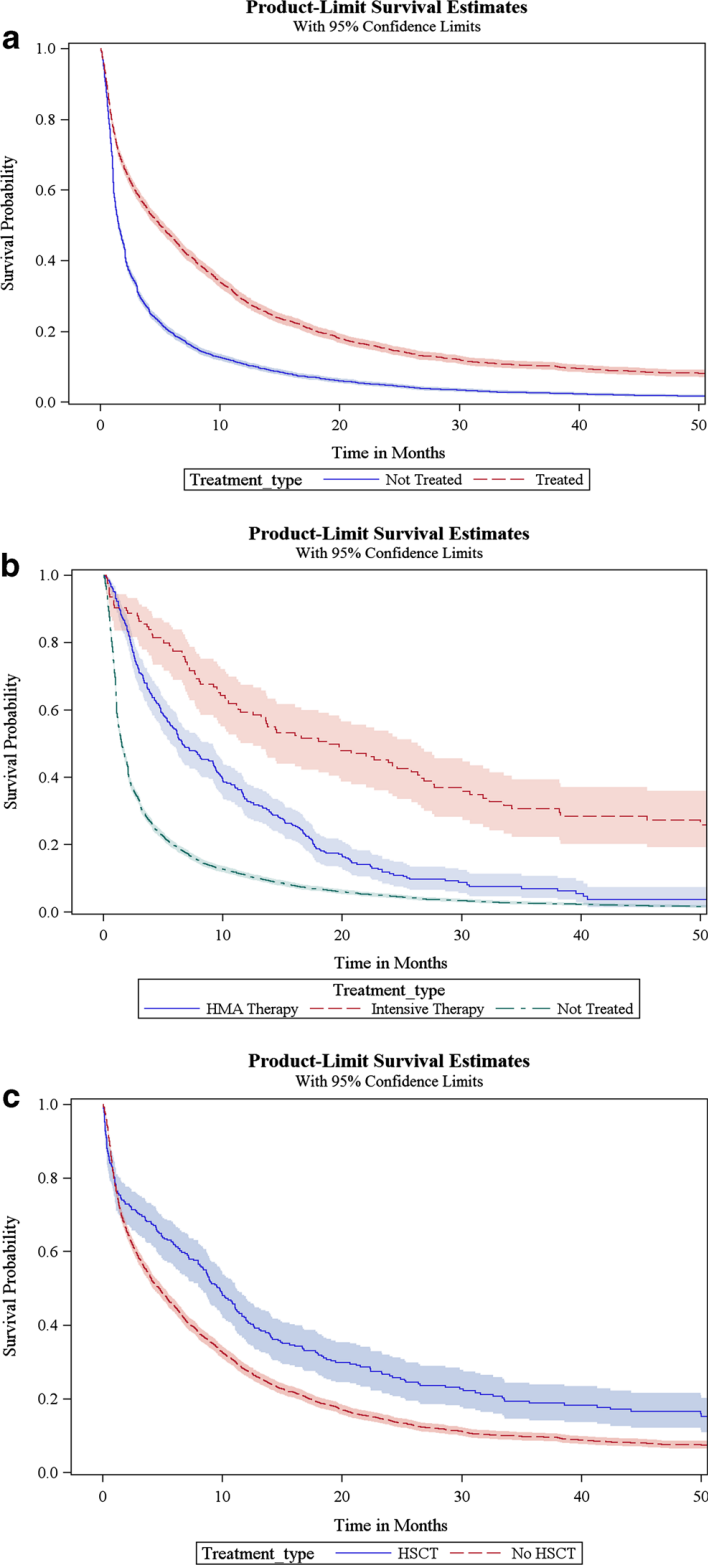
**a** Unadjusted overall survival by treatment status. **b** Unadjusted overall survival by treatment type. **c** Unadjusted overall survival among treated patients with and without HSCT

The median unadjusted overall survival was 2.5 months for the overall population and was longer for treated patients (5.0 months) compared to that for untreated patients (1.5 months; log rank *p* < .0001; Fig. 2a). As the observed overall survival was not significantly different between patients initiating therapy between 0–30 days, 30–60 days, and 60–90 days from diagnosis, all treated patients were analyzed as a single cohort. In multivariate survival analysis (Table 2), treated patients exhibited a 33 % lower risk of death compared to untreated patients (hazard ratio (HR) = 0.67; 95 % confidence interval (CI) = 0.64–0.71). Increasing age, increasing comorbidity score, and PPI were significantly associated with higher mortality risks. Prognosis also improved over time with a 7–12 % reduction in mortality during the years 2002–2004 (HR = 0.93; 95 % CI = 0.87–0.99), 2005–2007 (HR = 0.88; 95 % CI = 0.82–0.93), and 2008–2009 (HR = 0.90; 95 % CI = 0.84–0.97) compared to that during 2000–2001 (data not shown).

**Table 2:** Adjusted overall survival and risk of early death by treatment status

Covariates	All AML^a^ *N* = 8320			30-day mortality^a,b^ *N* = 7287		60-day mortality^a,b^ *N* = 7287	
	N	HR	95 % CI	HR	95 % CI	HR	95 % CI
Treatment status							
Not-treated (ref)	4999						
Treated	3321	0.67	0.64–0.71	0.34	0.29–0.39	0.56	0.51–0.61
Age at diagnosis							
66–70 (ref)	1320						
71–75	1771	1.29	1.19–1.39	1.19	0.98–1.45	1.37	1.19–1.56
76–80	1968	1.48	1.37–1.60	1.17	0.97–1.41	1.44	1.26–1.64
> 80	3261	1.79	1.66–1.93	1.38	1.16–1.65	1.62	1.43–1.84
Sex							
Male (ref)	4323						
Female	3997	0.96	0.91–1.01	1.02	0.92–1.13	1.02	0.95–1.10
Marital status							
Married (ref)	4369						
Widowed	2486	1.15	1.08–1.21	1.16	1.03–1.31	1.15	1.06–1.25
Separated/divorced	543	1.15	1.05–1.26	1.28	1.06–1.55	1.16	1.01–1.34
Single	532	1.16	1.06–1.27	1.20	0.99–1.47	1.25	1.09–1.43
Unknown	390	1.01	0.91–1.12	1.13	0.90–1.40	1.04	0.88–1.22
Prior MDS^c^							
No (ref)	6882						
Yes	1438	1.03	0.97–1.09	0.99	0.88–1.12	0.96	0.88–1.05
PPI^d^							
No (ref)	7270						
Yes	1050	1.28	1.20–1.38	1.32	1.16–1.50	1.25	1.13–1.37
NCI co-morbidity score							
0 (ref)	4257						
1	2103	1.15	1.09–1.22	1.09	0.96–1.22	1.13	1.04–1.23
2	1014	1.26	1.17–1.35	1.18	1.02–1.36	1.32	1.19–1.46
≥ 3	946	1.39	1.29–1.50	1.32	1.13–1.53	1.34	1.20–1.50

*NCI* National Cancer Institute, *MDS* prior myelodysplastic syndrome, *PPI* poor performance indicators, *HR* hazard ratio, *CI* confidence interval

^a^Model also includes race, geographic region, income, and year of diagnosis

^b^Treated group restricted to patients who received treatment within 30 days after diagnosis

^c^Patients with a prior myelodysplastic syndrome (MDS) or myeloproliferative disease was identified from Medicare claims and was used as a proxy for high-risk patients in the absence of disease stage

^d^Poor performance indicators(PPI) were identified from Medicare claims and include the use of oxygen and related respiratory therapy supplies, wheelchair and supplies, home health agency services, and skilled nursing facility services that occurred 12 months prior to AML diagnosis

We conducted exploratory subgroup analyses on the impact of treatment versus no treatment to examine whether specific prognostic subgroups benefited more or less from treatment (Supplementary Table S1). Receipt of treatment had a larger impact on mortality risk in the subgroup without a prior MDS (35 % reduction in mortality) compared to the subgroup with a prior MDS (20 % reduction in mortality). Treated patients in the younger age cohort, female gender, with presence of PPI, and NCI comorbidity score 1+ exhibited greater reductions in mortality compared to their subgroup counterparts. Marital status subgroups showed similar benefits with receipt of treatment.

The multivariate analysis of factors predicting early death is shown in Table 2. The median time to treatment initiation was 17 days. There were 1747 (24 %) of patients who died within 30 days of diagnosis and 3429 (47 %) that died within 60 days of diagnosis. Stratifying by treatment status, 210 (9 %) treated patients and 1537 (31 %) untreated patients died within 30 days of diagnosis. Treated patients had a 66 % lower likelihood of early death within 30 days of diagnosis and a 44 % lower likelihood of early death within 60 days of diagnosis compared to the untreated cohort. Other factors associated with increased risk of early death include older age, unmarried, higher comorbidity burden, and presence of poor performance indicators.

### Outcomes according to treatment modality

Patients receiving intensive therapy were younger (mean age 73 vs. 78 and 81), were more likely male (62 vs. 59 and 50 %), were married (71 vs. 61 and 47 %), had less secondary AML (7 vs. 21 and 19 % with prior MDS), were less likely to have PPIs (2 vs. 9 and 17 %), and had lower comorbidity score compared to those receiving HMA therapy and not treated, respectively (Table 3). Similarities in age, comorbidity burden, and proportion with high-risk disease were noted in HMA therapy and not treated patients. The median unadjusted overall survival (Fig. 2b) was longer for patients treated with intensive therapy (18.9 months) compared to that with HMA therapy (6.6 months) and not treated (1.5 months; log rank *p* < .0001). After adjusting for all covariates in the survival model, a 67 % reduction in mortality was observed among patients treated with intensive therapy and a 50 % reduction in mortality was observed among patients treated with HMA therapy, compared to not treated (Table 4). The propensity score-matched survival analysis demonstrated similar risk reductions for both intensive (62 % reduction in mortality) and HMA (59 % reduction in mortality) regimens compared to patients who were not treated. Increasing age, increasing comorbidity score, and presence of PPIs were associated with significant increases in mortality. In a subset analysis stratified by age, similar mortality risk reductions with receipt of intensive and HMA therapies were maintained in the younger (≤75) and older (>75) cohorts (data not shown).

**Table 3:** Baseline patient characteristics by type of treatment received

Characteristic	Not Treated *N* = 5009	HMA Therapy *N* = 345	Intensive Therapy *N* = 124	*p*	HSCT *N* = 276	No HSCT *N* = 3051	*p*
	%	%	%	%	%	%	
Age at diagnosis							
66–70	8.8	13.6	39.5	<0.0001	44.6	24.8	<0.0001
71–75	15.9	24.1	31.5		25.4	29.7	
76–80	23.3	25.5	16.1		14.5	25.0	
> 80	51.9	36.8	12.9		15.6	20.5	
Sex							
Male	49.9	59.1	62.1	0.0002	61.6	54.5	0.0228
Female	50.1	40.9	37.9		38.4	45.5	
Race/ethnicity							
White	87.2	90.4	87.9	0.2092	88.4	87.6	0.7118
Non-white	6.7	9.6	12.1		11.6	12.4	
Prior MDS^a^							
No	81.0	79.1	100^c^	0.0026	88.8	85.0	0.0920
Yes	19.0	20.9			11.2	15.0	
PPI^b^							
No	83.2	91.3	100^c^	<0.0001	94.2	93.4	0.6245
Yes	16.8	8.7			5.8	6.6	
NCI co-morbidity score							
0	47.3	50.7	55.6	0.1113	55.8	57.2	0.2711
1	25.2	25.8	25.8		22.8	25.5	
2	13.8	11.6	18.5^c^		10.9	9.7	
≥ 3	13.7	11.9			10.5	7.6	
Marital status							
Married	46.8	61.2	71.0	<0.0001	59.4	61.1	0.0851
Widowed	35.3	21.4	15.3		18.5	22.1	
Separated/divorced	6.5	5.5	13.6^c^		10.1	6.2	
Single	6.4	6.7			7.6	6.4	
Unknown	5.1	5.2			4.3	4.2	
% of adults with some college education							
0–50	42.8	35.7	45.2	0.0744	35.9	41.7	0.1610
51–100	52.7	58.0	54.8^c^		59.4	53.6	
Unknown	4.5	6.4			4.7	4.8	
Median income quartiles							
1–Low	26.2	21.2	24.2	0.0359	17.0	23.6	0.0419
2	25.2	21.2	30.6		25.7	24.5	
3	24.9	28.7	21.8		24.6	25.1	
4–High	23.5	29.0	23.4		32.6	26.6	
Geographic region							
Midwest	9.6	9.3	11.3	0.0305	12.7	11.2	0.1858
Northeast	6.0	7.2	10.5		4.0	6.7	
South	37.6	29.9	33.9		35.1	37.9	
West	46.8	53.6	44.4		48.2	44.2	

*HMA* hypomethylating agents, *HSCT* allogeneic hematopoietic stem cell transplantation, *NCI* National Cancer Institute, *MDS* prior myelodysplastic syndrome, *PPI* poor performance indicators

^a^Patients with a prior myelodysplastic syndrome (MDS) or myeloproliferative disease was identified from Medicare claims and was used as a proxy for high-risk patients in the absence of disease stage

^b^Poor performance indicators(PPI) were identified from Medicare claims and include the use of oxygen and related respiratory therapy supplies, wheelchair and supplies, home health agency services, and skilled nursing facility services that occurred 12 months prior to AML diagnosis

^c^Cells with counts of less than 11 are combined in compliance with the National Cancer Institute data use agreement for small cell sizes

**Table 4:** Adjusted overall survival by treatment type

Covariates	N	Multivariate cox regression^a^		Propensity score matched cox regression^b^	
Treatment		HR	95 % CI	HR	95 % CI
Not treated (ref)	5009				
HMA therapy	345	0.50	0.45–0.57	0.41	0.32–0.53
Intensive therapy	124	0.33	0.27–0.41	0.38	0.21–0.70
Age at diagnosis					
66–70 (ref)	537				
71–75	920	1.30	1.16–1.46		
76–80	1276	1.42	1.28–1.58		
> 80	2745	1.67	1.50–1.84		
Sex					
Male (ref)	2780				
Female	2698	1.01	0.95–1.07		
Marital status					
Married (ref)	2644				
Widowed	1859	1.13	1.05–1.20		
Separated/divorced	349	1.10	0.98–1.24		
Single	349	1.19	1.06–1.33		
Unknown	277	1.00	0.88–1.14		
Prior MDS^c^					
No (ref)	4445				
Yes	1033	0.98	0.91–1.05		
PPI^d^					
No (ref)	4605				
Yes	873	1.28	1.18–1.39		
NCI co-morbidity score					
0 (ref)	2611				
1	1383	1.17	1.10–1.25		
2	749	1.28	1.18–1.40		
≥ 3	735	1.37	1.26–1.50		

*HMA* hypomethylating agents, *NCI* National Cancer Institute, *MDS* prior myelodysplastic syndrome, *PPI* poor performance indicators, *HR* hazard ratio, *CI* confidence interval

^a^Model also includes race, geographic region, income, and year of diagnosis

^b^Propensity score matched for age, sex, race, marital status, education, geographic region, year of diagnosis, prior MDS, poor performance indicators, and comorbidity score

^c^Patients with a prior myelodysplastic syndrome (MDS) or myeloproliferative disease was identified from Medicare claims and was used as a proxy for high-risk patients in the absence of disease stage

^d^Poor performance indicators(PPI) were identified from Medicare claims and include the use of oxygen and related respiratory therapy supplies, wheelchair and supplies, home health agency services, and skilled nursing facility services that occurred 12 months prior to AML diagnosis

### Effect of allogeneic stem cell transplantation on survival

Among treated patients, there were 276 (8 %) who underwent HSCT therapy and 3051 (92 %) who did not (Table 3). HSCT patients were younger at diagnosis with mean age of 73 compared to the non-HSCT group (75 years; *p* < .0001). Seventy percent of HSCT patients compared to 55 % of non-HSCT patients were under the age of 75 at diagnosis. HSCT patients were also more likely to be male (62 vs. 55; *p* = 0.0228). There were no statistical differences in comorbidity burden, PPI, or prior MDS between both groups. Figure 2c shows that the unadjusted median overall survival was higher for HSCT (9.7 months) compared to the non-HSCT group (4.7 months; log rank *p* ≤ 0.0001). In multivariate survival analysis (Table 5), treated patients who underwent HSCT had a significant 21 % lower risk of death compared to those who did not receive HSCT. Increasing age, male gender, unmarried, prior MDS, PPI, and increasing comorbidity score were significantly associated with higher risks of post-treatment mortality. In an exploratory subset analysis stratified by age, the survival benefit with HSCT was only demonstrated in the younger age cohort ≤75 years old, and no difference in mortality risks was noted in the older age cohort >75 years (Table 5).

**Table 5:** Adjusted overall survival among treated patients with and without HSCT

Covariates	Treated^a^ *N* = 3321			≤75 years^a^ *N* = 1854		>75 years^a^ *N* = 1467	
Treatment	N	HR	95 % CI	HR	95 % CI	HR	95 % CI
No HSCT (ref)	3045						
HSCT	276	0.79	0.69–0.91	0.63	0.53–0.75	1.20	0.95–1.52
Age at diagnosis							
66–70 (ref)	880						
71–75	974	1.23	1.11–1.36				
76–80	802	1.50	1.35–1.66				
> 80	665	2.01	1.79–2.25				
Sex							
Male (ref)	1829						
Female	1492	0.88	0.82–0.95	0.83	0.75–0.93	0.96	0.85–1.08
Marital status							
Married (ref)	2025						
Widowed	724	1.16	1.05–1.28	1.23	1.06–1.43	1.16	1.02–1.31
Separated/divorced	218	1.26	1.08–1.46	1.20	0.99–1.44	1.27	0.98–1.65
Single	216	1.13	0.97–1.31	1.14	0.94–1.39	1.00	0.78–1.28
Unknown	138	1.03	0.86–1.24	1.04	0.81–1.33	1.10	0.84–1.44
Prior MDS^b^							
No (ref)	2834						
Yes	487	1.19	1.08–1.32	1.22	1.06–1.41	1.20	1.03–1.38
PPI^c^							
No (ref)	3107						
Yes	214	1.28	1.10–1.49	1.30	1.03–1.65	1.36	1.11–1.67
NCI co-morbidity score							
0 (ref)	1896						
1	841	1.14	1.05–1.25	1.17	1.04–1.32	1.13	1.00–1.29
2	323	1.22	1.07–1.38	1.18	0.98–1.42	1.22	1.01–1.46
≥ 3	261	1.42	1.24–1.64	1.42	1.17–1.73	1.40	1.14–1.73

*NCI* National Cancer Institute, *HSCT* allogeneic hematopoietic stem cell transplantation, *MDS* prior myelodysplastic syndrome, *PPI* poor performance indicators, *HR* hazard ratio, *CI* confidence interval

^a^Model also includes race, geographic region, income, and year of diagnosis

^b^Patients with a prior myelodysplastic syndrome (MDS) or myeloproliferative disease was identified from Medicare claims and was used as a proxy for high-risk patients in the absence of disease stage

^c^Poor performance indicators(PPI) were identified from Medicare claims and include the use of oxygen and related respiratory therapy supplies, wheelchair and supplies, home health agency services, and skilled nursing facility services that occurred 12 months prior to AML diagnosis

## Discussion

Although therapy use has increased over time, this large observational study of Medicare beneficiaries showed that currently, about 50 % of elderly AML patients remain untreated following diagnosis, which represents an unmet need. We observed a significant survival benefit with receiving anti-leukemic therapy, even among the HMA therapy group who had similar characteristics to the untreated patients. Further, improved survival after receiving intensive therapy compared to HMA therapy was noted after adjustment for confounding variables. However, when patients were matched on sex, race, marital status, education, prior MDS, PPI, and comorbidity score, we found mortality risk reductions of a similar magnitude with receiving both regimens. Overall, these real-world results provide further support that age alone should not deter the use of guideline-recommended therapies particularly because of the high disparities in outcomes between treatment receipt and palliative care.

Results from our observational study have been supported in prior RCTs involving elderly patients. Over 20 years ago, the European Organization for Research and Treatment of Cancer (EORTC) Leukemia Group demonstrated an improvement in complete remission rate and overall survival for AML patients aged 65 years or older immediately treated with induction chemotherapy compared to supportive measures only [27]. Significant clinical improvements in outcomes have also been demonstrated in elderly patients following HMA therapy. When compared to best supportive care (BSC) or low-dose cytarabine (LDAC), treatment with decitabine was associated with a significantly higher CR rate plus CRp rate, a trend toward improvement in median overall survival and a 20 % reduction in the risk of death [28]. In comparison, two separate studies compared the effects of azacitidine against conventional care regimens (CCRs) including BSC, LDAC, and conventional induction chemotherapy. In oligoblastic AML (<30 % blasts), azacitidine treatment was associated with significant improvements in median OS and 2-year survival, albeit no improvement in complete remission rate was observed [29]. In patients with >30 % blasts, preliminary reports showed a trend toward improvement in median OS, a 15 % reduction in the risk of death, improved 1-year survival, and no differences in the CR rate plus CRp rate [30]. No significant safety concerns were raised in these studies following HMA therapy.

Our results confirm data from other registry-based analyses that showed that elderly AML patients who received treatment exhibited a lower early death rate compared to untreated patients or palliation [11, 12, 31]. Although our multivariate analysis demonstrated a greater reduction in mortality in patients receiving aggressive induction chemotherapy compared to HMA therapy, both therapeutic options appeared to be equally better than supportive measures when the cohorts were properly matched for relevant cofounders.

Only 8 % of patients receiving chemotherapy underwent subsequent HSCT therapy. Chronologic age appears to be the driving factor in receiving HSCT. HSCT therapy was associated with a 20 % lower risk of death compared to patients receiving chemotherapy only, and the survival benefit was more pronounced among the younger cohort (≤75 years) with a 37 % reduction in mortality risk. Although myeloablative allogeneic HSCT is rarely recommended in older patients with significant comorbidities, reduced-intensity conditioning (RIC) allogeneic HSCT is encouraging when used as post-remission therapy [9, 10, 32]. The NCCN guidelines consider RIC allogeneic HSCT an additional option for patients 60 years or older as post-remission therapy in those who achieved complete response from induction therapy [7]. Although our observations are at best hypothesis generating, they raise the question of whether allogeneic HSCT provides therapeutic benefit to AML patients older than 75 years of age. Prospective and well-controlled clinical trials are needed to define the role of allogeneic HSCT as post-remission therapy in this cohort of patients.

In the current study, receipt of treatment varied by gender, marital status, income, and geographic region, similar to patterns observed in prior oncology research [33–35].Our results also demonstrate that married AML patients were more likely to receive therapy and had higher survival compared to unmarried patients, even after adjusting for known confounders [35]. These results highlight the importance of marital status, likely as a surrogate of social-economic support in patients with AML, and confirm results from previous reports focusing on solid tumor malignancies. Further research is warranted to better quantify how nonclinical factors such as social support contribute to receipt of cancer therapy and outcomes.

The finding that patients receiving intensive therapy were younger, were more likely male, were married, had less secondary AML, were less likely to have PPIs, and had lower comorbidity score compared to those receiving HMA therapy and no treatment may reflect a belief among physicians that elderly patients are frailer and less able to tolerate aggressive or more toxic treatments [4, 36–38]. These observations are in agreement with previously reported patterns of treatment selection. For example, in two recent randomized trials where pre-selection of CCR was performed prior to randomization, subjects assigned to aggressive treatment modalities were a median of 5–8 years younger than their counterparts assigned to less intensive regimens [29, 30]. Elderly patients also have diverse attitudes toward cancer treatment; some desire aggressive treatment modalities while others decline therapies offered by their oncologist [39, 40]. These age disparities in treatment patterns are associated with higher mortality [4, 5], and our results provide further support that demographic factors such as age should not discourage the use of guideline-recommended therapies.

### Strengths and limitations

Use of the SEER-Medicare data for this type of analysis has several strengths, including the large sample size from a population-based registry and the diverse geographic representation of AML patients in the USA. The database includes longitudinal data with claims for covered services from the time a person is eligible for Medicare until the date of death regardless of residence or service area.

The results of the comparative effectiveness analysis should be interpreted with caution due to the large amount of missing data and resulting small sample size of treatment groups. Induction chemotherapy with curative intent in the outpatient setting is applied to very select elderly AML patients, and our findings may not be representative of the general patient population receiving intensive induction therapy. Conventional chemotherapy treatments for AML are highly toxic [8] and generally requires inpatient treatment. Inpatient stays are paid based on ICD-9 diagnosis or procedure codes only, and therefore, we were unable to define the type of chemotherapy received for 70 % of the treated cohort without the specific chemotherapy J code. Further, dose selection was at the discretion of the physician and dosing information could not be determined retrospectively from available data within the claims dataset.

The SEER registry does not collect baseline molecular and cytogenetic information for leukemia, and our surrogate for stage (including claims for prior MDS as a marker of disease severity) may not adequately assess stage in all patients in our study. The SEER-Medicare data did not contain remission status prior to HSCT, and type of prior anti-AML therapy was not known for the majority of patients receiving transplant. In addition, the SEER data does not include measures of performance status, and using Medicare claims to identify several indictors of poor performance may also be subject to bias. Performance status influences clinicians’ decisions to treat or the specific regimen to administer. Information regarding treatment patterns and characteristics of patients enrolled in health maintenance organizations (HMOs) or fee-for-service plans was not available since Medicare does not collect these data. Treatment patterns, prognosis, and complications may differ between these alternative health care plans and Medicare enrollees, and this would be a productive area for additional evaluation.

## Conclusion

Overall, these real-world results provide further support that age alone should not deter the use of guideline-recommended therapies in AML. Our results highlight the benefit of treatment in contrast to palliative therapy in this underserved patient population of elderly AML patients and suggest that anti-leukemic regimens should be strongly considered in the majority of older patients. But, even with treatment, outcomes remain dismal, and given this important unmet medical need, many new agents are currently in development for older patients with AML [41–44]. Our findings provide an important context for therapeutic selection that occurs in older patients with AML in the USA. Moving forward, it will be important to identify patients less likely to be treated at diagnosis and design clinical trials to address the therapeutic challenges that exist in this cohort of patients.

## Electronic supplementary material

Below is the link to the electronic supplementary material.

Supplementary Table S1 (DOCX 16 kb)
