# Supplementary material for: Big data analysis of treatment patterns and outcomes among elderly acute myeloid leukemia patients in the United States
Source: Ann Hematol. 2015 Mar 20;94(7):1127–38. doi: 10.1007/s00277-015-2351-x (PMC4432101; doi:10.1007/s00277-015-2351-x)
Supplement: Supplementary file 1 — (DOCX 16 kb) [file 277_2015_2351_MOESM1_ESM.docx]

**Supplementary Table 1: Risk of death among Treated vs. Not-Treated in different prognostic subgroups**

| Covariates | Treatment Status | All AML ^a^  N=8320 | | |
| --- | --- | --- | --- | --- |
|  |  | N | HR | 95% CI |
| Age at Diagnosis |  |  |  |  |
| ≤ 75 | Not-treated | 1237 | ref |  |
|  | Treated | 1854 | 0.63 | 0.58-0.68 |
| >75 | Not-treated | 3762 | ref |  |
|  | Treated | 1467 | 0.68 | 0.63-0.71 |
| Sex |  |  |  |  |
| Male | Not-treated | 2494 | ref |  |
|  | Treated | 1829 | 0.69 | 0.65-0.74 |
| Female | Not-treated | 2505 | ref |  |
|  | Treated | 1492 | 0.65 | 0.60-0.70 |
| Marital Status |  |  |  |  |
| Married | Not-treated | 2344 | ref |  |
|  | Treated | 2025 | 0.67 | 0.62-0.72 |
| Unmarried | Not-treated | 2655 | ref |  |
|  | Treated | 1296 | 0.67 | 0.62-0.72 |
| Prior MDS ^1^ |  |  |  |  |
| No | Not-treated | 4048 | ref |  |
|  | Treated | 2834 | 0.65 | 0.61-0.69 |
| Yes | Not-treated | 951 | ref |  |
|  | Treated | 487 | 0.80 | 0.70-0.90 |
| PPI ^2^ |  |  |  |  |
| No | Not-treated | 4163 | ref |  |
|  | Treated | 3107 | 0.68 | 0.64-0.71 |
| Yes | Not-treated | 836 | ref |  |
|  | Treated | 214 | 0.61 | 0.52-0.73 |
| NCI Co-morbidity Score |  |  |  |  |
| 0 | Not-treated | 2361 | ref |  |
|  | Treated | 1896 | 0.70 | 0.65-0.75 |
| 1+ | Not-treated | 2638 | ref |  |
|  | Treated | 1425 | 0.63 | 0.58-0.68 |

Abbreviations: NCI, National Cancer Institute; MDS, prior myelodysplastic syndrome; PPI, poor performance indicators

^1^ Patients with a prior myelodysplastic syndrome (MDS) or myeloproliferative disease was identified from Medicare claims and was used as a proxy for high risk patients in the absence of disease stage.

^2^ Poor performance indicators (PPI) were identified from Medicare claims and include the use of oxygen and related respiratory therapy supplies, wheelchair and supplies, home health agency services, and skilled nursing facility services that occurred 12 months prior to AML diagnosis

^a^ All models adjusted for all other variables on the table as well as race, geographic region, income and year of diagnosis
